# Comparison of Common Respiratory Virus Peak Incidence Among Varying Age Groups in Rhode Island, 2012-2016

**DOI:** 10.1001/jamanetworkopen.2020.7041

**Published:** 2020-05-13

**Authors:** Young June Choe, Michael A. Smit, Leonard A. Mermel

**Affiliations:** 1 Department of Pediatrics, Warren Alpert Medical School of Brown University, Providence, Rhode Island; 2 Department of Social and Preventive Medicine, Hallym University College of Medicine, Chuncheon, South Korea; 3 Division of Infectious Diseases, Children’s Hospital Los Angeles, Los Angeles, California; 4 Keck School of Medicine, University of Southern California, Los Angeles; 5 Department of Epidemiology and Infection Control, Division of Infectious Diseases, Rhode Island Hospital, Providence; 6 Department of Medicine, Warren Alpert Medical School of Brown University, Providence, Rhode Island

## Abstract

This case series study assesses the seasonality of respiratory viral infections among different age groups in Rhode Island from 2012 to 2016.

## Introduction

Although children and young adults appear to drive the spread of influenza virus and respiratory syncytial virus (RSV) in community settings,^1,2,3^ this issue remains unclear.^4^ We assessed the seasonality of respiratory viral infections with respect to age in 1 locale.

## Methods

The study was approved by the Rhode Island Hospital institutional review board and was granted a waiver of informed consent because it involves the use of secondary data. This study follows the Strengthening the Reporting of Observational Studies in Epidemiology (STROBE) reporting guideline.

This cross-sectional case series involved detection of respiratory viruses among different age groups in Rhode Island. Data were derived from respiratory specimens sent to the Lifespan Microbiology Laboratory from inpatients and outpatients assessed at a Lifespan facility, which was licensed for 1165 beds and 757 380 outpatient visits in 2017, as well as patient specimens from other outpatient facilities throughout Rhode Island. All respiratory viruses positively identified in the laboratory by use of the respiratory viral panel and rapid influenza or RSV tests were reviewed. Adenovirus, coronavirus (not coronavirus disease 2019), influenza, parainfluenza, RSV, and human metapneumovirus were included for analysis. The weekly number of positive tests between January 2012 and December 2016 was extracted to construct a longitudinal case series for 4 age groups: 0 to 4 years, 5 to 17 years, 18 to 64 years, and 65 years and older.

Analyses were performed using R Studio statistical software version 1.2.503 (R Project for Statistical Computing). Data analysis was performed from December 2018 to April 2019. Time series analysis and cross-correlation analysis were applied to determine the detection of respiratory viruses in the age group with the highest incidence and its correlation with detection in different age groups. Statistical significance was set at *P* < .05 and was calculated with linear regression and 2-sided Fisher exact tests.

## Results

A total of 6733 respiratory viruses were detected and were predominantly detected in the following weeks: adenovirus, multiple peaks; coronavirus, weeks 50 to 7 (560 of 677 cases [83%]); influenza virus, weeks 50 to 9 (1487 of 1825 cases [84%]); parainfluenza virus, multiple weeks; RSV, weeks 48 to 11 (2198 of 2278 cases [97%]); and human metapneumovirus, weeks 1 to 17 (950 of 1017 cases [93%]) (Figure). With the exception of influenza virus, which first started in the 18- to 64-year age group, most respiratory virus infections occurred in the 0- to 4-year age group before other age groups, including peak detection of the coronavirus (Table). The Table shows the number and percentage of total cases, the lagged period (in weeks) with respect to the reference age group (ie, 0-4 years for adenovirus), and their mean (SE) correlation coefficients with statistical significance. In the 65 years and older age group, peak detection of influenza virus, parainfluenza virus, and human metapneumovirus occurred 3 weeks after peak detection in the 0- to 4-year age group (mean [SE] correlation coefficient, 0.215 [0.064]; *P* = .001), 4 weeks after peak detection in the 5- to 17-year age group (mean [SE] correlation coefficient, 0.155 [0.044]; *P* < .001), and 2 weeks after peak detection in the 18- to 64-year age group (mean [SE] correlation coefficient, 0.139 [0.038]; *P* < .001), respectively. Peak detection of RSV in the 65 years and older age group occurred 4 weeks after peak detection in the 0- to 4-year age group (mean [SE] correlation coefficient, 0.079 [0.021]; *P* < .001).

**Figure.**
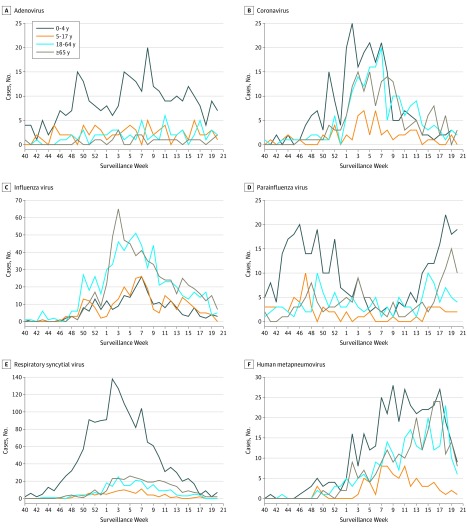
Seasonality of Respiratory Viruses Among Different Age Groups Graphs show number of cases of infection by surveillance week for adenovirus (A), coronavirus (B), influenza virus (C), parainfluenza virus (D), respiratory syncytial virus (E), and human metapneumovirus (F).

**Table.** Cross-Correlation Between Age Groups in Detection of Respiratory Viruses

Virus and age group, y	Total cases, No. (%)	Lag period after detection in the reference group, wk	Correlation coefficient, mean (SE)	*P* value
Adenovirus (n = 582)				
0-4	358 (61.5)	[Reference]		
5-17	103 (17.7)	10	0.112 (0.033)	.001
18-64	89 (15.3)	1	0.076 (0.031)	.02
≥65	32 (5.5)	1	0.036 (0.016)	.02
Coronavirus (n = 677)				
0-4	254 (37.5)	[Reference]		
5-17	59 (8.7)	4	0.070 (0.027)	.01
18-64	192 (28.4)	5	0.152 (0.040)	<.001
≥65	172 (25.4)	5	0.147 (0.045)	.001
Influenza virus (n = 1825)				
0-4	249 (13.6)	1	0.153 (0.027)	<.001
5-17	271 (14.8)	1	0.188 (0.033)	<.001
18-64	665 (36.4)	[Reference]		
≥65	640 (35.1)	3	0.215 (0.064)	.001
Parainfluenza virus (n = 1036)				
0-4	496 (47.9)	[Reference]		
5-17	95 (9.2)	1	0.053 (0.022)	.02
18-64	220 (21.2)	1	0.089 (0.035)	.01
≥65	225 (21.7)	4	0.155 (0.044)	<.001
Respiratory syncytial virus (n = 2278)				
0-4	1612 (70.8)	[Reference]		
5-17	104 (4.6)	4	0.023 (0.010)	.02
18-64	237 (10.4)	4	0.067 (0.016)	<.001
≥65	325 (14.3)	3	0.079 (0.021)	<.001
Human metapneumovirus (n = 1017)				
0-4	430 (42.3)	[Reference]		
5-17	77 (7.6)	1	0.085 (0.022)	<.001
18-64	252 (24.8)	1	0.170 (0.041)	<.001
≥65	258 (25.4)	2	0.139 (0.038)	<.001

## Discussion

We found that the detection of RSV in the youngest age group, for whom the incidence first peaked, was correlated with its detection in older age groups weeks later. This finding was similar to that of a Belgian study^4^ that found peak RSV detection in elderly patients (aged ≥65 years) 6 weeks after peak detection in young children (aged <6 years). In contrast, a study from Hong Kong^3^ found no age synchrony, suggesting that geographic, climactic, and/or cultural differences (eg, mixing between different age groups or social interactions) may play a role in determining the epidemiology of RSV. A Canadian study^1^ found that peak influenza activity occurred in the 10- to 19-year age group before other age groups. A study^5^ of social contact networks suggests that this may reflect the high number of contact level hours among high school students being the local transmission driver of influenza. However, we identified peak detection of influenza virus initially in adults aged 18 to 64 years. It is unclear whether this reflects differences in past exposure to the influenza virus strains circulating during our study period,^6^ or whether there is another yet unidentified explanation. We also found that, like other respiratory viruses, coronavirus first peaked among young children aged 0 to 4 years and then was followed by a peak among children aged 5 to 17 years. It is important to learn the transmission dynamics of coronavirus to guide policy making on public health countermeasures for the current coronavirus 2019 pandemic.

The cases analyzed in our study were all medically attended illnesses, which do not reflect asymptomatic cases or all mild cases in the community. Moreover, detection bias is possible because health care practitioners may have been more likely to test for respiratory virus detection in children compared with adults. Despite these limitations, our study sought to identify the relationships of seasonality of multiple respiratory viruses in different age groups. Future studies are needed to develop a conceptual model for age-specific epidemiology of respiratory viruses and their interactions within the population.
